# Mesenchymal Stem Cell-Conditioned Medium Modulates Apoptotic and Stress-Related Gene Expression, Ameliorates Maturation and Allows for the Development of Immature Human Oocytes after Artificial Activation

**DOI:** 10.3390/genes8120371

**Published:** 2017-12-08

**Authors:** Hakimeh Akbari, Seyed Hassan Eftekhar Vaghefi, Abbas Shahedi, Victoria Habibzadeh, Tooraj Reza Mirshekari, Aboozar Ganjizadegan, Hamidreza Mollaei, Meysam Ahmadi, Seyed Noureddin Nematollahi-Mahani

**Affiliations:** 1 Department of Anatomy, Afzalipour Faculty of Medicine, Kerman University of Medical Sciences, 7616913555 Kerman, Iran; ghsuper@sums.ac.ir or anaakbari91@gmail.com (H.A.); nnematollahi@kmu.ac.ir (S.N.N.-M.); 2 Cellular and Molecular Research Center, Gerash University of Medical Science, 7441758666 Gerash, Iran; 3 Department of Biology and Anatomical Sciences, Shahid Sadoughi University of Medical Sciences, 8916978477 Yazd, Iran; 4 Afzalipour Clinical Center for Infertility, Afzalipour Hospital, Kerman University of Medical Sciences, 7616913555 Kerman, Iran; V.habibzadeh@gmail.com (V.H.); mirshekari@kmu.ac.ir (T.R.M.); agivf20@gmail.com (A.G.); 5 Department of Medical Microbiology, Kerman University of Medical Sciences, 7616913555 Kerman, Iran; hamid2008kmu@gmail.com; 6 Neuroscience Research Center, Institute of Neuropharmacology, Kerman University of Medical Sciences, 7616913555 Kerman, Iran; meysamcell@yahoo.com

**Keywords:** conditioned medium, gene expression, oocytes, mesenchymal stem cells, in vitro oocyte

## Abstract

The aim of the present study was to determine whether mesenchymal stem cell-conditioned medium (MSC-CM) modulates apoptotic and stress-related gene expression, and ameliorates maturation and developmental potential of immature human oocytes after artificial activation. A total of 247 surplus immature germinal vesicle (GV) oocytes obtained from infertile women were allocated into two in vitro maturation (IVM) groups: 1: GV oocytes (*n* = 116) matured in vitro (fIVM), and 2: GV oocytes (*n* = 131) that were vitrified, then in vitro matured (vIVM). Also, two maturation media were used: Alpha-minimum essential medium (α-MEM) and human umbilical cord-derived MSCs (hUCM). After 36 h of incubation, the IVM oocytes were examined for nuclear maturation. In IVM-matured oocytes, cytoplasmic maturation was evaluated after artificial activation through Ionomycin. Moreover, the quantitative expressions of B-cell CLL/lymphoma 2 (*BCL2*), BCL2-associated X protein (*BAX*), superoxide dismutase (*SOD*), and Heat shock proteins (*HSP70*) in matured oocytes were assessed by quantitative Real-time polymerase chain reaction (qRT-PCR) and compared with fresh and vitrified in vivo matured oocytes, which were used as fIVM and vIVM controls, respectively. The highest maturation rate was found in hUCM in fIVM, and the lowest maturation rate was found using α-MEM in vIVM (85.18% and 71.42%, respectively). The cleavage rate in fIVM was higher than that in vIVM (83.4% vs. 72.0%). In addition, the cleavage rate in α-MEM was lower than that in the hUCM (66.0% vs. 89.4%). Furthermore, the difference between parthenote embryo arrested in 4–8 cells (*p* < 0.04) and the quality of embryo arrested in 8-cell (*p* < 0.007) were significant. The developmental stages of parthenote embryos in hUCM versus α-MEM were as follows: 2–4 cell (89.45% vs. 66.00%, respectively), 4–8 cell (44.31% vs. 29.11%, respectively), morula (12.27% vs. 2.63%, respectively), and blastocysts (2.5% vs. 0%, respectively). The messenger RNA (mRNA) expression levels of BCL2, BAX and SOD were significantly different (*p* < 0.05) between the matured IVM oocytes. Overall, hUCM showed potential efficacy in terms of ameliorating oocyte maturation and in promoting the development and mRNA expression of BAX, BCL2, and SOD.

## 1. Introduction

Oocyte cryopreservation and the in vitro maturation (IVM) of immature oocytes are vital techniques for female fertility preservation in assisted reproductive technology (ART). However, meiotic spindle damage and the risk of aneuploidy in embryos are increased in the cryopreservation of mature oocytes. In the germinal vesicle (GV) stage, chromatin is carefully enveloped in the nucleus, decreasing the risk of meiotic spindle damage [1]. Approximately 20% of the retrieved oocytes are immature following controlled ovarian hyper-stimulation, and immature oocytes are routinely discarded. The IVM of immature oocytes may prevent further cycles, thereby reducing the cost and negative side effects of using gonadotropins. Furthermore, oocyte cryopreservation is useful in fertility preservation, especially in countries with ethical limitations regarding the freezing of embryos [2,3]. The success rate of maturation in vitrified immature oocytes seems to be higher than that of maturation in vitrified mature oocytes. However, vitrified immature oocytes need to be thawed prior to IVM [4,5]. IVM is a cheap method for fertility preservation in patients with cancer or polycystic ovary syndrome (PCOS) [6]. Different IVM media have been introduced for oocyte development. However, in spite of significant developments in IVM media, rates of fertilization and live births are unsatisfactory [1,6]. During IVM, nuclear maturation occurs, but cytoplasmic maturation is not completed. Therefore, introducing an ideal IVM medium that mimics in vivo follicular actions has been the topic of previous research [7]. In an IVM cycle, reaching cytoplasmic maturation is as important as reaching nuclear maturation [1]. The maturation process is crucial in this technique, and the choice of the ideal medium for human IVM is considered particularly difficult [8]. In vitro parthenogenesis can be prompted by chemical, mechanical, or electrical stimulation and is a direct indicator in the assessment of cytoplasmic oocyte development [9]. Furthermore, in oocyte development, numerous cytokines and growth factors operate by autocrine and paracrine secretions [10]. Various mesenchymal stem cell-conditioned media (MSC-CM) have been used in many different types of diseases and have been proven to improve healing and treatment. Supernatants of MSCs contain numerous growth factors, cytokines, bioactive factors, and tissue regenerative agents; they are secreted from MSCs, and can modulate IVM and subsequent embryonic development [11,12]. In vitro investigations have shown that human umbilical cord MSCs (hUCM) actively impede the functions of numerous immune cells through secreted growth factors and cytokines. Similarly, CM supernatants derived from hUCM might be useful in culture media through their amino acids, cytokines, and vitamins, all of which affect serum components [12,13]. It is known that growth factors stimulate meiotic progress; also, meiotic progress is important in IVM processes, which may help adjust the mRNA expression in oocytes, oocyte maturation and cytoplasmic development following in vitro fertilization (IVF). Moreover, oocyte quality is vital in embryonic development during fertilization; however, some oocytes are arrested at the metaphase II (MII) prior to fertilization. Furthermore, prolonging the in vitro culture time would have adverse impact on future oocyte development [14,15]. BCL2-associated X protein (*BAX*) is a pro-apoptotic gene that stimulates cell death. B-cell CLL/lymphoma 2 (*BCL2*) is an anti-apoptotic gene that stimulates cell survival [16]. The main intracellular scavenger enzyme of reactive oxygen species (ROS) is Cu/Zn superoxide dismutase (SOD), which is located in the mitochondria. Damaged mitochondria alter the microenvironment for oocytes, reducing the quality, fertilization rate and developmental competence of oocytes [17]. Heat shock proteins (HSPs) are critical stress proteins in reproduction and are molecular chaperones for the regulation and protection of cellular function and structure [18]. Growth factors and cytokines stimulate meiotic development and IVM processes. Although the paracrine effects of MSCs have been described, human oocyte protection using hUCM secretions has never been researched. Until now, no studies have been performed on the messenger RNA (mRNA expression levels of genes related to human oocyte vitrification and IVM in hUCM. The aim of the current study was to determine whether MSC-CM can modulate apoptotic and stress-related gene expression, ameliorate maturation and developmental potential of immature human oocytes after artificial activation.

## 2. Materials and Methods

### 2.1. Study Design

This experimental study involved GV oocytes (*n* = 247) obtained from 117 patients (30.5 ± 5.6 years old) who were referred to the Infertility Center of Afzalipour Hospital in Kerman. This study was approved by the ethics committee of Kerman University of Medical Sciences (*n*: 93–692) in March 2015 (IRCT2017101736840N1). Written informed consent was obtained from all the participants. This study complies with the Patient’s Bill of Rights, as immature oocytes, which are not useful clinically, were used. Criterion for female inclusion was: age lower than 35 years old; and the exclusion criteria were as follows: endometriosis, PCOS, abnormal chromosome, low ovarian response, chronic anovulation and fewer than five oocytes on retrieval. Fresh and vitrified GV oocytes were used, and IVM was randomly performed in two conditioned media for 36 h; then, all oocytes were assessed for maturity under an inverted microscope. Matured oocytes were evaluated for cytoplasmic maturity by studying the oocyte activation and cleavage competence of the oocytes.

### 2.2. Oocyte Collection

Oocyte collection was done 36 h after the injection of 10,000 IU of Human chorionic gonadotropin (hCG) (IBSA, Lugano, Switzerland) through laparoscopy. After denudation using 80 IU of hyaluronidase (Sigma, St. Louis, MO, USA) and mechanical dissection with pipetting, the denuded oocytes were examined for nuclear maturity. According to the extrusion of first polar body (PB), the oocytes were labeled MΙΙ or immature according to GV breakdown (GVBD). Germinal vesicle oocytes were also collected for this study [19].

### 2.3. Vitrification

First, the GV oocytes were suspended in an equilibration solution containing 7.5% ethylene glycol (EG) (Merck, Darmstadt, Germany) and 7.5% dimethyl sulfoxide (DMSO) (Merck) in Ham’s F10 medium supplemented with 20% human serum albumin (HSA) (Plasbumin, Clayton, NC, USA) at room temperature for 5–15 min. After, the oocytes were submerged in vitrification solution containing 15% EG and 15% DMSO with 0.5 M sucrose (Sigma) in Ham’s F10 complemented with 20% HSA for 50–60 s at room temperature. Then, the oocytes were instantly placed on a Cryotop (Kitazato, Kyoto, Japan). These samples were moved to a liquid nitrogen storage tank for 2 months [3].

### 2.4. Thawing

The oocytes were thawed in a five-stage thawing solution; 1: (1 M sucrose in Ham’s F10 supplemented with 20% HSA) for 50–60 s; 2: dilution solution 1 (0.5 M sucrose in Ham’s F10 supplemented with 20% HSA) for 3 min; 3: dilution solution 2 (0.25 M sucrose in Ham’s F10 supplemented with 20% HSA) for 3 min; and 4/5: washing solutions 1 and 2 (Ham’s F10 supplemented with 20% HAS) for 3–5 min each. Viability of the vitrified oocytes was assessed using a stereomicroscope 2–3 h after thawing; alive oocytes were placed in IVM medium in an incubator for 36 h [20].

### 2.5. In Vitro Maturation Media

Two media were used. Medium I was α-minimum essential medium (α-MEM) and was used as a control medium [21]. Medium II was hUCM [22].

### 2.6. MSC Isolation and Culture

Human umbilical cord MSCs were obtained from the Cell Culture Laboratory of Afzalipour Medical University in Kerman, Iran. These cells were positive in MSC surface markers clusters of differentiation (or CD) molecules (CD44, CD73, CD90, and CD105). The hUCM were differentiated into osteocytes, adipocytes, and neural cells [23]. After washing these cells with phosphate-buffered saline (PBS), they were incubated in MSC growth medium containing α-MEM supplemented with 10% FBS, 100 U/mL of penicillin, and 100 mg/mL of streptomycin. After 3 days, no adherent cells were detached in 2–3 washes with 1x PBS, and adherent cells were cultured further in the medium until they reached 90% confluence. The medium transformed once; after 48 h, the supernatants of these cells were collected, and with 0.2-µm membranes were filtered [24].

### 2.7. Artificial Oocyte Activation

Mature oocytes in the IVM medium were stimulated for parthenogenesis using ionomycin (Sigma) and 6-dimethylaminopurine (6-[DMAP]) (Sigma). Then, α-MEM, as a control medium, was supplemented with 10 µM of ionomycin at 37 °C for 6 min. After, the oocytes were moved to an IVF culture medium (Vitrolife, Gothenburg, Sweden) supplemented with 2 µM of 6-DMAP and maintained at 6% CO_2_ and 37 °C for 3 h. Next, the activated oocytes were washed twice and transferred into 30 mL droplets of fresh cleavage medium (Vitrolife) under equilibrated mineral oil in a humidified atmosphere of 5% CO_2_ at 37 °C. The developmental potential of the stimulated oocytes was maintained for 6 days after activation [25].

### 2.8. Scoring of Parthenote Embryos

The morphology of parthenote embryos is scored by size, blastomere number, and cytoplasmic fragmentation on a scale from A–C, with A being the highest quality with equally symmetrical blastomeres and minor fragmentation (lower than 10%), B being moderate quality with equally symmetrical blastomeres and moderate fragmentation (25–30%), and C being poor quality with blastomeres of unequal sizes and severe cytoplasmic fragmentation )more than 50%) [26].

### 2.9. RNA Extraction and cDNA Synthesis

RNA was extracted from a 100 μL volume of sample using a precipitation method (RIBO-prep, ILS, Moscow, Russia). Briefly, specimen was added to a tube containing 300 μL of lysis buffer [Tris-Hcl, TritonX100, SDS (sodium dodecyl sulfate), and Tween20] and 400 μL of precipitate buffer, mixed, they incubated for 10 min at room temperature; centrifuge in 13,000 rpm for three minutes. After this washed once with 500 μL of wash buffer W3, once with 200 μL of 70% (*v*/*v*) ethanol (Wash buffer W4), and then dried at 60 °C for 10 min. It was then resuspended in 50 μL of RNase-free water and converted into complementary DNA (cDNA) by *Real-time* polymerase chain reaction (RT-PCR). For Reverse Transcription 5 μL of RNA was added to a reaction mixture (15 μL) containing 20 mM Tris-HCl (pH 8.4), 50 mM KCl, 7.5 mM MgCl_2_, each deoxynucleotid triphosphate at a concentration of 1.5 mM, 25 ng of each random primer [(pdN)6, 1.6 U of RNasin, and 200 U of Moloney murine leukemia virus reverse transcriptase (Thermofisher, Darmstadt, Germany). The reaction mixture was incubated at room temperature for 10 min, 37 °C for 45 min, and 95 °C for 5 min and quenched on ice [27].

### 2.10. qRT-PCR

Real time PCR primers were designed for target genes mRNAs after alignment of these regions between all of them in The European Bioinformatics Institute (EMBL*-*EBI) database. Glyceraldehyde-3-phosphate dehydrogenase (*GAPDH*) purchased from Metabion in Germany was used as an internal control. qRT-PCR was carried out on a Corbett Rotor-Gene 6000 (Qiagen, Germantown, MD, USA). The transcript abundance of *SOD*, *HSP70*, *BCL2*, and *BAX* was analyzed by qRT-PCR. The raw data were then analyzed with the Relative expression software tool (REST) version 2.2.3 (Qiagen) using the automatic cycle threshold (Ct) setting to assign baselines and thresholds for the Ct determination. Delta Ct (ΔΔCT) values were used for this analysis. The relative expressions (REs) of the sample genes were calculated using the ΔΔCT method and *GAPDH* was used as the internal control or housekeeping gene.

### 2.11. Statistical Analysis

The differences were calculated using a chi-square test, and the Kruskal–Wallis test was used to obtain nonparametric data. The significance was examined using SPSS Statistics version 21 (IBM, Armonk, NY, USA); *p* values less than 0.05 were considered to be significant.

## 3. Results

No significant differences existed in the demographic characteristics of the patients included in the studied groups, including their etiology of infertility (*p* < 0.432), age (*p* < 0.756) and infertility duration (*p* < 0.227). Fresh GV oocytes (*n* = 116) and live GV oocytes after thawing (*n* = 116/131) were cultured in these IVM media. The viability of the vitrified oocytes was evaluated after thawing; their survival rate was found to be 88.54%. In total, 116 GV oocytes (of the original 131) were in vitro matured. The GV oocytes were divided into two groups: fresh IVM (fIVM) and vitrified IVM (vIVM) randomly cultured in α-MEM or hUCM media.

### Oocyte Maturation after IVM

The oocyte maturation rate was reduced in vIVM compared to fIVM in both media. In the vIVM group, the oocyte maturation rate was more reduced in α-MEM (71.4%) than in hUCM (79.2%). In the fIVM group, the oocyte maturation rate was higher in hUCM than in α-MEM (85.1% vs. 72.5%). The total maturation rate (in both fIVM and vIVM oocytes) in hUCM was significantly higher than that in α-MEM (82.21% vs. 72%) (*p* < 0.000) (Table 1). There was a significant difference between the GVBD arrest (*p* value < 0.036) between the groups (Table 1).

During oocyte maturation, GV oocyte nuclei must be broken down, and their chromosomes must be condensed, in the metaphase of the first meiotic division stage (MI); but MII oocytes are mature and have polar body (PB). Within the columns, the oocyte maturation rate and GVBD arrest differed significantly (*p* < 0.05) according to the chi-square test.

**Figure 1:**
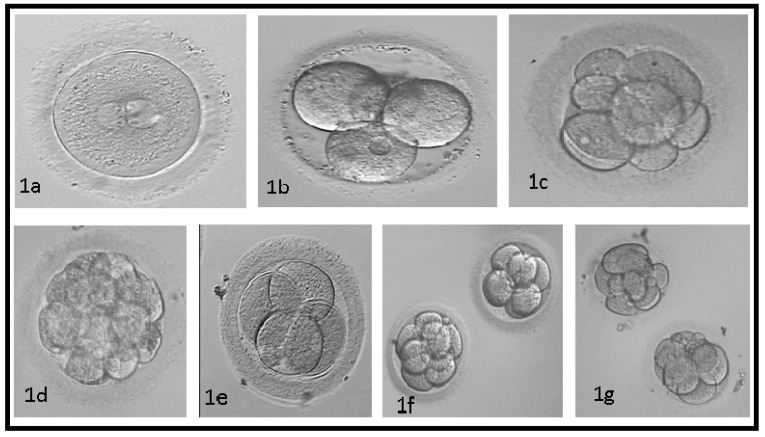
Microscopic observation of the cleavage rate of activated oocytes. About 11–18 h after mature oocyte activation, the fertilization pronucleus (**a**) develops; about 30 h into the activation process, the first embryonic cell is formed. 2–4 cells embryo, were formed on the second day after fertilization (**b**); and 4–8 cells embryo, were formed on the third day after fertilization (**c**). Compaction occurred 68 h after activation (**d**); expanded blastocysts formed on day five after oocyte fertilization (through activation or insemination). Parthenote embryos were scored according to the A, B, and C scale described above (**e**–**g**); the embryo formation of 2–4 cells was scored as A (**e**); the embryo formation of 4–8 cells was scored as B (**f**); and the embryo formation of 4–8 cells was scored as C (**g**).

For the assessment of the cytoplasmic maturation of MII oocytes (matured in conditioned IVM medium), the developmental competence of in vitro matured oocytes was evaluated through chemical activation (Figure 1) [28].

Embryo formation in the activated oocytes was assessed after parthenogenesis. The percentage of activated oocytes, the percentage of two pronuclei (2PN), the percentage of degenerated oocytes, the cleavage rate, the parthenote embryo scores, and blastocyst development were compared between groups (Table 2).

Activated oocytes are mature MII oocytes whose parthenogenesis is stimulated by ionomycin. Degenerated oocytes are activated oocytes that do not develop 2PN. The parthenote embryos scores were evaluated with the highest quality parthenote embryos given a score of A and the lowest quality parthenote embryos given a score of C.

The differences between 4–8 cells embryo being arrested (*p* < 0.04) and the A score of 4–8 cells embryo (*p* < 0.007) were both significant (Table 2).

After oocyte activation, several oocytes developed 2PN, but some did not and then degenerated. The rate of degenerated oocytes in α-MEM in fIVM compared to that in hUCM in fIVM were 24% and 9.09%, respectively, and that in α-MEM in vIVM compared to that in hUCM in vIVM were 44% and 12%, respectively. We compared the percentage of embryo formation in each developmental stage (2–4 cell, 4–8 cell, morula, and blastocysts). Embryos that were able to reach higher levels of cell division were recorded to be in lower stages than their development, as the process of development is gradual. Therefore, each of the 4–8 cell embryos, the 2–4 cell embryos, and the morula were registered as a 2–4 cell embryo formation or 4–8 embryo formation, as they passed these stages. Furthermore, each blastocyst passed all the embryo developmental stages (2–4 cell, 4–8 cell, and morula). The percentage of 2–4 cell embryo formation was calculated according to the number of activated oocytes, and the percentages of 4–8 cell embryo formation and 16-cell embryo formations were calculated according to the number of cleaved embryos. The developmental stages of activated oocytes, in α-MEM in fIVM compared to those in hUCM in fIVM were as follows: 2–4 cell embryo (76.0% vs. 90.9%, respectively), 4–8 cell embryo (36.8% vs. 75.0%, respectively), morula formation (5.2% vs. 20.0%, respectively), and blastocysts formation (0% vs. 5%, respectively). The developmental stages of activated oocytes in α-MEM in vIVM compared to those in hUCM in vIVM were as follows: 2–4 cell embryo (56% vs. 88%, respectively), 4–8 cell embryo (21.4% vs. 13.6%, respectively), and morula formation (0% vs. 4.5%, respectively); neither experienced blastocyst formation (Table 3).

According to our study, the highest cleavage rate was found in fIVM MSCs, in which 5% of the activated oocytes reached the blastocyst stage. No significant differences were found between the groups in terms of the rates of 2–4 cell embryo formation (0.4), 4–8 cell embryo (0.6) and 16 cell embryo formation (0.7). 

The primer sequences used for the qRT-PCR are shown in Table 4.

**Figure 2:**
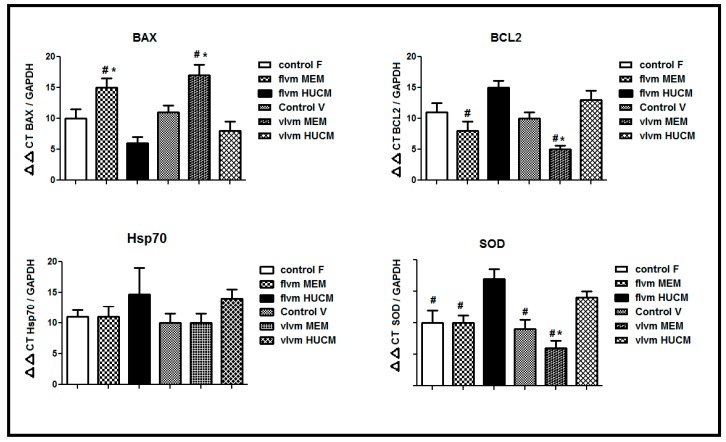
Expression of apoptotic genes (*BAX* and *BCL2*) and stress genes *(HSP70* and *SOD*) in IVM oocytes. Each value represents the mean ± standard error of the mean (SEM) of 17 oocytes per group. The IVM groups were compared with the control groups (fresh and vitrified in vivo matured oocytes obtain from cancelled intracytoplasmic sperm injection (ICSI). 1: Control F represents the fresh in vivo matured oocytes; 2: Control V represents the vitrified in vivo matured oocytes; 3: fIVM: Fresh GV oocytes were placed in α-MEM or hUCM and then matured; 4: vIVM: vitrified GV oocytes were placed in α-MEM or hUCM and then matured; * represents a significant difference (*p* < 0.05) with the hUCM in vIVM group; #: represents a significant difference (*p* < 0.05) with the hUCM in fIVM group.

Four IVM groups were compared with the two control groups of in vivo matured human oocytes: Control V (in vivo matured oocytes that vitrified) and Control F (fresh in vivo matured oocytes). In vivo matured oocytes were obtained from patients that cancelled their intracytoplasmic sperm injection (ICSI) on the puncture day of oocyte retrieval; these were prepared in the control groups for apoptotic and stress gene evaluation using a method approved by the ethics committee of our institute. The inclusion and exclusion criteria for this study are described in the Materials and Methods section. In the present study, the mRNA expression levels of BCL2 and BAX (both apoptotic genes) and those of SOD and HSP70 (both stress genes) within in vitro matured oocytes were significantly different (*p* < 0.05); except for HSP70, in the vIVM and fIVM groups in α-MEM and hUCM. The expression of HSP70 mRNA showed no significant difference between the groups. Conversely, the REs of BCL2 mRNA showed significant differences between α-MEM in vIVM and α-MEM in fIVM with hUCM in fIVM. Further, α-MEM in vIVM was significantly different than hUCM in vIVM. The REs of BAX mRNA showed significant differences between α-MEM in fIVM and α-MEM in vIVM with hUCM in vIVM and hUCM in fIVM. The REs of SOD mRNA showed significant differences between Control F, Control V, α-MEM in fIVM, and α-MEM in vIVM with hUCM in fIVM. Furthermore, α-MEM in vIVM was significantly different with hUCM in vIVM. The RE of HSP70 mRNA showed upregulation in hUCM in vIVM and hUCM in fIVM; this was not significantly different between groups (Figure 2). The highest expression of BCL2 was observed in hUCM in fIVM, and the lowest expression of BCL2 was observed in α-MEM in vIVM. The lowest expression of BAX was detected in hUCM in fIVM, and the highest expression of BAX was detected in α-MEM in vIVM. The ratio of BAX to BCL2 is an indicator of prolonged maturity; the highest BAX/BCL2 ratio was observed in α-MEM in vIVM (3.4), and the lowest BAX/BCL2 ratio was observed in hUCM in fIVM (0.4). The ratio of BAX to BCL2 was increased in the vitrified oocytes (Figure 2). The highest relative amount of SOD was observed in hUCM in fIVM, and the lowest relative amount of SOD was observed in α-MEM in vIVM. The mRNA expression of HSP70 in MSC-CM was higher than that of α-MEM in vitrified oocytes and in the fIVM group (Figure 2).

## 4. Discussion

One of the main problems related to mature oocyte cryopreservation, is the sensitivity of the meiotic spindle toward cryoprotectants and low temperature. Then, oocyte cryopreserving in the GV stage, due to the chromatins, are enveloped by nuclear membrane [29]. Consequently, combining the vitrification of GV oocytes and IVM offers an effective technique for fertility preservation and the development of oocyte banks [29]. There is a low efficiency of current commercial IVM media and their costs are high; moreover, potential therapeutic hUCM paracrine mechanisms can be harnessed using hUCM as an IVM medium. The components of culture media and IVM conditions, affect mRNA content, the proteomic profile, and oocyte maturation through cellular and molecular processes [30]. In this study, the oocyte maturation rate was higher in fIVM compared to that in vIVM. This is similar to the findings of Shahedi et al. [31]; however, the oocyte maturation rate in our study was higher, which may be caused by variations in the IVM media. Our results also agree with Ling et al., who found that the maturation rate of mouse oocytes was higher in MSC-CM compared to that in α-MEM (91.2% vs. 63.5%, respectively) [24]; these differences with our data may be caused by differences in the cell culture techniques, MSC, and oocyte sources used. Meanwhile, in vitro matured oocytes generally have minor developmental capacity, but the complete maturation of oocytes is essential for ensuring the competence of embryos [32]. The loss of developmental capacity is related to the lack of specific proteins in oocytes cultured in vitro to MII [32]. In this study, the cleavage rate in fIVM was higher than in vIVM; this is similar to the findings of Cobo et al. [33], who concluded that Cryotop vitrification was the safest method for oocyte cryopreservation, as it protects developmental capacity [33]. The developmental rate of oocytes in our study was lower than that in Cobo et al., which may be caused by differences in the type of IVM media used in each study [34]. Peters et al. indicated that the rate of blastocyst formation of MSCs was higher than in mouse embryonic fibroblast medium (95.1% vs. 91.7%, respectively) [35]. They also described that low embryonic developmental rates may be associated with oocyte quality in nuclear and cytoplasmic maturation; this is affected by the components of culture media. As such, the enrichment of MSC-CM may be useful to improve embryonic developmental rates. The differences in the results of Peters et al. and our results might be related to differences in vitrification, thawing, and IVM, all of which lead to oocyte injury throughout these processes [35]. Acton et al. noted that the developmental rate of embryos cultured in KSOM Embryo Culture was higher than that of embryos cultured in human tubal fluid (HTF), as the mitochondrial distribution in embryos cultured in KSOM Embryo Culture is higher than that in HTF [36]. In our study, the highest amount of embryonic arrest occurred on day three after artificial activation. Changing the medium was required to resupply nutrients, which may be due to the lack of nutrients in the medium [36]. In contrast to our study, in the study by Acton et al., the culture medium was changed to Gardner’s G2 medium on day three after fertilization simultaneous with the formation of the 4–8 cell embryo [36]. In the same way, Cobo et al. changed the culture medium on the third day after fertilization in their study [33]. Embryos arrested in early stages and poor developmental rates are both related to the culture medium, because the medium, could not provide regular nuclear and cytoplasmic development for oocytes; thus, the attempt to enrich IVM medium could be beneficial for oocyte maturation and development [37]. The method of fertilization and the components of culture media could also change mitochondrial distribution; moreover, mitochondrial structure and function must be studied in pre-implantation development [36]. de Fried et al. reported the parthenogenesis activation rate (86.1%), cleavage rate (96.8%), and blastocyst rate (16.7%), all of which were higher than the rates outlined in our results [25]. This may be caused by the differences in culture media or the use of mature vitrified oocytes without IVM during maturation. The developmental competence of oocytes influenced by chromosomal arrangement, cytoplasmic organelle delivery, maternal mRNA, and critical proteins for embryonic development [1]. Matured oocytes have several proteins and embryonic mRNA that protect embryonic development, but arrest can occur at any developmental stage during in vitro culturing, from the zygote stage to the blastocyst stage. Numerous investigations have described that the high expression of *BCL2* is related to good-quality oocytes [38]. Embryonic development can be influenced by the functional balance of apoptosis and cellular proliferation. Furthermore, the *BCL2* gene family plays a main role in apoptotic pathways [38]. When maturation is prolonged, the ratio of *BAX* to *BCL2* increases significantly; thus, BCL2 and BAX are key in evaluating oocyte aging [39]. In this study, the highest increase in the ratio of *BAX* to *BCL2* was observed in α-MEM in vIVM. The manipulation of oocytes during vitrification and IVM induces high levels of ROS [40]. Superoxide dismutase, acts as an antioxidant gene in cumulus cells. The high expression of SOD has been detected in mature human oocytes. In porcine oocytes, the high expression of SOD reduces DNA damage and promotes fertilization and development in oocytes [41]. In our study, the best rate of embryonic development was observed in MSCs in fIVM; these showed a higher expression of SOD when compared to the control. In addition, HSPs can protect oocytes against oxidative stress. Some studies have described that the expression of HSP is reduced in the vitrified thawing of MII oocytes; this may be the main cause of decreased oocyte viability and rates of development in vitrified oocytes [42,43]. In vIVM oocytes, the high expression of SOD might be a protection mechanism against osmotic and heat stress during vitrification [44]. Nevertheless, Molina et al. proved that the osmotic shock produced during vitrification improves the rate of oocyte maturation, embryo cleavage, and intracellular calcium (Ca^+2^) concentration [45]. In the present study, the mRNA levels of BAX, BCL2, and SOD were significantly different between groups due to vitrification and the different IVM media used, both of which may alter ROS production and energy metabolism [28]. Maternal inherited mitochondria, provide energy to blastomeres; also, the number of mitochondria and the number of cristae could have major effects on embryonic development [36]. Blastomere mitochondria are derived from oocytes, as sperm mitochondria are degenerated during fertilization; thus, a significant correlation exists between oocyte mitochondria and embryonic development [36]. Mitochondria are randomly distributed during mitosis; hence, blastomeres with inadequate numbers of mitochondria might be fragmented, or malfunctions may occur during development due to low ATP levels [46]. Also, the alterations of zona pellucida and mitochondria-smooth endoplasmic reticulum (M-SER) aggregates in vitrified oocytes, probably reduce the embryo formation and cleavage rates [47]. The number of mitochondria in mature oocytes is higher than that in immature mitochondria. Furthermore, mitochondria have major effects in oocyte fertilization and development through the regulation of free calcium and the manufacturing of ATP in cells [46]. The association between mitochondria and smooth endoplasmic reticulum complexes could lead to calcium homeostasis. Therefore, a low fertilization rate is likely associated with variations in the M-SER complexes during vitrification. Finally, oocyte mRNA expression may predict subsequent embryonic development [31].

## 5. Conclusions

The maturation and cleavage rate of activated oocytes were both improved in MSC-CM. The relative expressions of *BAX*, *BCL2*, and *SOD* in IVM oocytes seem to be associated with oocyte development competence; in addition, hUCM is likely to generate a microenvironment that is more appropriate for inducing oocyte maturation and increasing the development of embryos than α-MEM.

## Figures and Tables

**Table 1:** The effect of conditioned media on oocyte maturation rate.

Type of IVM	Number of IVM GV Oocyte	Arrest in GV Stage *n* (%)	Arrest in GVBD Stage *n* (%)	Number of MII Oocyte	Oocyte Maturation Rate
**fIVMα-MEM**	62	7 (11.29%)	10 (15.74%)	45	72.58%
**vIVMα-MEM**	63	15 (23.8%)	3 (4.76%)	45	71.42%
**fIVMhUCM**	54	7 (12.96%)	1 (1.85%)	46	85.18%
**vIVMhUCM**	53	4 (7.54%)	7 (13.2%)	42	79.24%
***p* value**	0.439	0.534	0.036	0.091	0.000

GV: germinal vesicle; IVM: in vitro maturation; fIVM: fresh GV oocyte, in vitro maturation; vIVM: vitrified GV oocyte, in vitro maturation; α-MEM: α-minimum essential medium; hUCM-CM: human umbilical cord mesenchymal stem cells conditioned medium; GV arrest: Oocyte arrested at the germinal vesicle stage; GVBD arrest: oocyte arrested at germinal vesicle breakdown (GVBD) stage; MII: Mature oocyte at metaphase II (MII) stage.

**Table 2:** The effect of conditioned media on developmental competence of in vitro matured oocytes.

Developmental Parameter	Group (1) fIVM in α-MEM	Group (2) vIVM in α-MEM	Group (3) fIVM in MSCs	Group (4) vIVMinMSCs	*p* Value
**MII oocyte (*n*)**	45	45	46	42	0.091
**Total activated oocyte (*n*)**	25	25	22	25	0.453
**Degenerated of activated oocyte (*n*)**	6	11	2	3	0.163
**2 Pronuclei (*n*)**	19	14	20	22	0.059
**Arrest in 2–4 cell (*n*)**	12	11	5	19	0.063
**Score A (2–4)**	7	5	4	11	0.243
**Score B (2–4)**	3	4	1	6	0.238
**Score C (2–4)**	2	2	-	2	0.584
**Arrest in 4–8 cell (*n*)**	6	3	11	2	0.044
**Score A (4–8)**	3	-	7	2	0.007
**Score B (4–8)**	3	2	3	-	0.352
**Score C (4–8)**	-	1	1	-	0.551
**Arrest in 16 cell (*n*)**	1	-	3	1	0.357
**Blastocyst (*n*)**	-	-	1	-	0.266

**Table 3:** The effect of conditioned media on human oocyte development after parthenogenesis activation.

IVM Groups	Activated Oocyte	Degenerated Oocyte after Activation	2–4 Cell * Formation	4–8 Cell ** Formation	16 Cell ** Formation	Blastocyst ** Formation
**fIVMα-MEM**	25	6 (24%)	19 (76%)	7 (36.8%)	1 (5.26%)	-
**vIVMα-MEM**	25	11 (44%)	14 (56%)	3 (21.42%)	-	-
**fIVM MSCs**	22	2 (9.09)	20 (90.9%)	15 (75%)	4 (20%)	1 (5%)
**vIVM MSCs**	25	3 (12%)	22 (88%)	3 (13.63%)	1 (4.54%)	-

* Percentage from the number of activated oocytes; ** Percentage from the number of cleaved embryos; Activated oocyte: In vitro matured oocytes were activated with ionomycin; Degenerated oocyte: activated oocytes, which couldn’t 2PN developed.

**Table 4:** Primers used in the quantitative reverse transcription PCR (RT-qPCR).

Gene Name	Primer Sequence (5′–3′ Orientation)	Product Size (bp)	Gene Bank Accession No.
***Bax***	Forward: TGGACAGTAACATGGAGC	143	NM_001291429
	Reverse: TGGCAAAGTAGAAAAGGG		
***Bcl2***	Forward: TGGCCTTCTTTGAGTTCG	108	NM_000633
	Reverse: TGCCGGTTCAGGTACTCAG		
***Hsp70***	Forward: TCGTGGAGGAGTTCAAGAG	172	NM_005345
	Reverse: GGTGATGGACGTGTAGAAG		
***SOD1***	Forward: AAAGATGGTGTGGCCGATGT	164	NM_000454
	Reverse: AGCCAAACGACTTCCAGCG		
***GAPDH***	Forward: GAAGGTGAAGGTCGGAGTC	221	NM_001289746
	Reverse: AAGATGGTGATGGGATTTC		

GAPDH: glyceraldehyde-3-phosphate dehydrogenase; BAX: BCL2-associated X protein; BCL2: B-cell CLL/lymphoma 2; SOD: superoxide dismutase.
